# A qualitative examination of the current management of opioid use disorder and barriers to prescribing buprenorphine in a Canadian emergency department

**DOI:** 10.1186/s12873-021-00443-1

**Published:** 2021-04-15

**Authors:** David Wiercigroch, Patricia Hoyeck, Hasan Sheikh, Jennifer Hulme

**Affiliations:** 1 Faculty of Medicine, University of Toronto, 1 King’s College Circle, Toronto, Canada; 2 Department of Family and Community Medicine, University of Toronto, Toronto, Canada; 3 University Health Network Emergency Department, Toronto, Canada

**Keywords:** Buprenorphine, Opioid withdrawal, Suboxone

## Abstract

**Background:**

Emergency departments (EDs) across Canada are increasingly prescribing buprenorphine for opioid use disorder (OUD). The objective of this study was to identify the current knowledge, attitudes, and behaviours of ED physicians on the management of OUD in the ED, including barriers and facilitators to prescribing buprenorphine.

**Methods:**

We purposefully selected emergency physicians from one ED in Toronto which had recently received education on OUD management and had a new addiction medicine follow-up clinic, to participate in semi-structured interviews. We used semi-structured interviews to explore experiences with patients with OUD, conceptions of role of the ED in addressing OUD, and specifically ask about perceptions and experience on using buprenorphine for opioid withdrawal. Our analysis was informed by constructivist grounded theory to help uncover contextualized social processes and focus on what people do and why they do it. Two researchers independently coded transcripts using an iterative constant comparative and interpretative approach.

**Results:**

Results fell broadly into facilitators and barriers. Generally, management of OUD in the ED varied significantly. Physician-level facilitators to treating opioid withdrawal with buprenorphine included: knowledge about OUD an7d buprenorphine, positive experiences with substitution therapy in the past, and the presence of physician champions. Systems-level facilitators included timely access to follow-up care and pre-printed order sets. Barriers included provider inexperience, lack of feedback on treatment effectiveness, limited time to counsel patients, and pressure to discharge patients quickly. Additional barriers included concerns about precipitating withdrawal, prescribing a chronic medication in acute care, and patient attitudes.

**Conclusion:**

This study describes barriers and facilitators to addressing OUD and prescribing buprenorphine in a Canadian ED. These findings suggest a role for additional provider education, involvement of allied health professionals in counseling, and mentorship by physician champions in the department.

**Supplementary Information:**

The online version contains supplementary material available at 10.1186/s12873-021-00443-1.

## Background

Opioid Use Disorder (OUD) is a problematic pattern of opioid use that results in impairment or distress of clinical significance [1]. OUD is a cause of significant and increasing harm, with 14,700 opioid-related deaths in Canada between January 2016 and September 2019 [2]. During this time period, 1 in 4 patients seen in EDs for opioid-related poisonings were admitted, resulting in 19,490 hospitalizations [2]. Since opioid-users frequently present to the ED in withdrawal, following an overdose, or with complications of injection drug use, it is an important opportunity to initiate treatment for OUD.

A recent Canadian clinical guideline recommends the use of the opioid agonist medication buprenorphine-naloxone (trade name Suboxone), from here on referred to as buprenorphine, as first-line treatment for OUD [3]. Buprenorphine offers advantages over methadone, including a ceiling effect which limits the abuse potential and risk of diversion, reduced stigma, and milder withdrawal symptoms [4, 5]. Buprenorphine is increasingly accessible in Ontario after being included on the Ontario Drug Benefit formulary [6]. Buprenorphine induction in the ED has been shown to be a feasible and effective approach to begin medication for addiction treatment [7–9]. A randomized clinical trial found that ED-initiated buprenorphine significantly increased engagement in addiction treatment and decreased use of inpatient addiction services compared to referral or brief interventions in the ED [7]. Retention on buprenorphine treatment has been shown to reduce ED utilization [10].

Widespread adoption of new practices takes time and has particularly lagged in the treatment of substance use disorders [11]. Previous studies have examined attitudes of primary care providers towards prescribing buprenorphine [12–15] as well as those of emergency physicians [16–18]. Yet there is limited literature on attitudes, knowledge and behaviours surrounding OUD and initiating buprenorphine in Canadian EDs [16]. We sought to explore Canadian emergency physician attitudes and experiences in order to inform communications and quality improvement strategies during the opioid crisis and help support patients and providers alike as buprenorphine prescribing expands across Canada.

To achieve this objective, we used grounded theory, a systematic qualitative methodology which is used to generate theories about social processes, experiences, and interactions [19, 20]. A constructivist approach to grounded theory recognizes that there can be multiple perspectives of reality which are shaped by outcomes, experiences, and interpretation within a changing context [21]. Constructivism also draws on researchers’ expertise and experiences to inform data collection and analysis. Constructivist grounded theory is increasingly used to understand health care processes, particularly in nursing research [22].

## Methods

The objective of this study was to assess the attitudes, motivation, experiences, and practices in the treatment of OUD in an ED in Toronto, Ontario. Our qualitative study design was informed by constructivist grounded theory to allow us to generate theory rooted in the data [20], while remaining reflexive during data collection. The research team consisted of two medical trainees with health policy backgrounds (DW, PH) and two academic emergency and addictions physicians (JH and HS). This approach thus allowed us to acknowledge our assumptions throughout, and that data analysis is a co-constructed process [21, 23].

### Sampling

We purposefully sampled ED physicians from one urban academic teaching hospital in Toronto, Ontario. This department was chosen as a department ‘in transition’ which had some exposure to OUD management: all physicians had received educational rounds on OUD management, an introduction to an opioid withdrawal order set, and the hospital had established a rapid-access addictions clinic for follow-up. We randomly invited half of the physicians from that group of eighty physicians to participate. We performed a simple randomization process using a random number generator to minimize selection bias. We limited invitations to half of the department’s physicians to ensure that all interviews could be completed within the study period.

### Data collection

We drafted and field tested a flexible semi-structured interview guide with topic experts, and modified interview questions to ensure developing categories were explored.

The guide consisted of open-ended questions about clinicians’ experiences with patients with opioid use disorder. We prompted if needed on their conceptions of the role of the ED in addressing opioid addiction, preventing future overdoses, and counselling around opioid use, treatment options, and harm reduction. The final portion elicited clinicians’ experiences and perceived barriers to prescribing buprenorphine, their training, interest, and resources required to facilitate prescribing buprenorphine. The interview guide is available for reference [see Additional file 1].

The first author (DW) conducted semi-structured interviews in person and over the phone based on participant preference between July and September 2018. Data collection and analysis continued until we reached theoretical thematic saturation after 19 participant interviews. Interviews ranged from fifteen to forty-five minutes in length. We recorded and transcribed the interviews verbatim, using pseudonyms during transcription to ensure anonymity. We obtained informed verbal consent from all participants and maintained confidentiality. The University Health Network Research Ethics Board approved the protocol as part of a quality improvement project to improve the care of patients with OUD.

### Data analysis

Constructivist grounded theory guided a step-wise approach to simultaneous data collection and analysis [21]. The first four transcripts were reviewed independently by DW, JH and HS to develop familiarity with the data and consensus on a broad coding framework. Two reviewers (DW, PH) independently organized and coded the transcripts through multiple readings to identify meaningful patterns, using an iterative constant comparative and interpretative approach. DW and PH reviewed the themes to ensure representativeness of the data and to reach a consensus on discordant views. A third reviewer (JH) reviewed and triangulated the thematic analysis with the two main reviewers. Data analysis was organized using Dedoose software (version 8.0.35, SocioCultural Research Consultants LLC, Los Angeles, CA).

## Results

Nineteen physicians participated in the study. Participants were predominantly male (*n* = 12; 63%) Almost half of participants (*n* = 9) were in their first five years of practice, five were mid-career having practiced for 6–20 years, and five had been in practice for over 20 years. Almost half (n = 9; 47%) were family physicians with additional training in emergency medicine (CCFP-EM), six were Royal College trained in emergency medicine (FRCP), and four were family physicians (CCFP).

Predominant concepts and themes are summarized here, with supportive quotes outlined in Tables 1, 2, and 3. Results were grouped under broad categories of barriers and facilitators to providing evidence-based treatment of opioid use disorder in the ED. Overall, we found wide variation in the current approaches to treating OUD in the ED. Barriers to treating OUD and prescribing buprenorphine in the ED included physician reports of limited knowledge and experience, inadequate nursing support, safety concerns, patient factors, and logistical challenges. Facilitators to managing opioid withdrawal included the presence of physician champions and positive experiences with opioid agonist therapy, as well as system-level factors such as the availability of order sets and timely access to follow-up for patients.

**Table 1:** Participant quotes supporting variability in OUD management of OUD

1. **Variable management of opioid withdrawal** i. “I think it’s a much more nuanced decision-making process to deal with withdrawal than with a patient who is acutely intoxicated and not breathing, someone you can use naloxone with as an intervention.” (Physician 12) ii. “I have a lot less experience treating people with opioid withdrawal than with an overdose and that I find can be extremely frustrating.” (Physician 19) iii. “There are some physicians who just simply take more time in terms of providing patient education and some who do less of that because of time restraints. There’s also variability in terms of physician interest in the subject matter. There’s variability in terms of what physicians feel should be done about these issues and what they feel their role is in the whole realm of addictions.” (Physician 4) iv. “I think more and more in terms of habits, people are comfortable using [buprenorphine].” (Physician 12) v. “There are a variety of different medications that you can use to deal with these people when they’re leaving. There’s low doses of clonidine, NSAIDs and laxatives. Before you discharge them, you want to sure that people are comfortable enough to leave. It depends on what kind of symptoms they’re having, whether or how quickly you’re able to discharge them.” (Physician 13) vi. “Clonidine is something that people have been using for a long time and using acetaminophen and naproxen to treat other symptoms of withdrawal. I think those things people are comfortable with and have been using for quite a while. I suspect there’s probably some people that give opiates and some people that give a lot of benzodiazepines, but I don’t. Some people, they give nothing and say no one dies of opiate withdrawal. I think it probably varies a lot.” (Physician 19) vii. “As a group we’ve decided we would not like to prescribe narcotics. I think there’s still a large number of other individuals that take other stances. Oh well, it’s Friday. Oh well, this person’s a nuisance. I’m just going to write whatever it is and give it to them and get them out of the department. I think that still goes on.” (Physician 8) viii. “Opioid withdrawal is not terribly successful. We don’t have the same medications as we do for alcohol withdrawal and I don’t think they’re effective.” (Physician 15) ix. “We never would have given them naloxone kits. We never would have given the buprenorphine. So all that is pretty new.” (Physician 13)

**Table 2:** Participant quotes supporting facilitators to treating withdrawal and initiating buprenorphine

1. **Departmental champions support physician knowledge** i. “I did not have formal training. Before [physician] came along and started working in our department, I never used it. I was aware it existed, but I never had any exposure. Once [physician] came along, they provided some guidelines on paper and gave some presentations to the group.” (Physician 1) ii. “I’ve never started it before, but I would be quite happy to start it now that there’s a order set that I can use and other colleagues that I work with that I can ask about it.” (Physician 5) iii. “I was able to prescribe [buprenorphine] with [physician] supporting me on What’sApp.” (Physician 19) iv. “You know it’s easy to approach [physician] and say, ‘here’s a situation I had, what do you think I could have done with that?’ So that is how I got my exposure.” (Physician 1) v. “[Physician] has also given us follow up stories, success stories essentially, of people who have followed up in the clinic and have significantly decreased their use of opioids. I found that very helpful.”(Physician 6) 2. **Physician empowerment & patient satisfaction** i. “I can describe two scenarios where the patient went from uncomfortable … just kind of “suffering through it” withdrawal where they know it is going to get worse … they went from feeling awful to smiling. One of them just shook my hand before leaving. He tracked me down and said thank you and then left, which is not necessarily part of my interaction with patients. It’s above and beyond to see that reaction in a patient. There are very few patients that feel that great when they leave the ED. I think it’s been quite positive.” (Physician 10) ii. “I don’t know what the outcomes are, I’ve heard they are good but the patient satisfaction when they leave the ED with an almost near resolution of their symptoms after one or two doses in most cases … it seems to be a positive from what I see.” (Physician 11) iii. “I’m giving someone who has a substance abuse issue a benzodiazepine and potent hypertensive medication to just go home with. I think they were never fully satisfied, and I was never satisfied with the interaction.” (Physician 7) iv. “It’s kind of fatiguing as a doctor to be like “No, I can’t fix this, I can’t fix that. We don’t do anything with this.” It is the helpless and useless which we do not like feeling. And so now that there is a tool in our hands, it has inspired us a bit. I feel this collectively in hearing how other people deal with patients. I think people feel a little bit more inspired to engage and to problem solve with patients, to counsel and positively support them … because we feel like we have something instead of nothing.” (Physician 19) 3. **Order sets** i. “Now that we have the order set, it makes it so much easier. It empowers people to use it because if someone looks at this, there are very straightforward including inclusion criteria, exclusion criteria. It’s become a lot easier to operationalize this knowledge not having to look it up on third party sources, about the proper dosing, the proper COWS threshold for instance, just all this stuff that we have had to find in other places. To have the institution and the Department behind a reviewed order set certainly empowered even myself. I think it has been a big change.” (Physician 12) ii. “We’ve got a good explanation hand out for patients and a good walk through for a physician with dosing suggestions and how to assess withdrawal. Given that it wasn’t part of my training formally, I’ve really found it helpful to have that resource.” (Physician 10) 4. **Timely access to follow-up care** i. “If there was no follow up for the patients, I wouldn’t go prescribing buprenorphine.” (Physician 2) ii. “It’s great when patients come in Monday evening, you can treat them accordingly and all they have to do is show up at a [follow-up clinic] at 9 am and an addiction specialist and a team will see them. I think that’s a great message to send to patients and its a great follow up plan that we have.” (Physician 12) iii. “If I’m sending patients with opioid withdrawal on [buprenorphine] on a Friday evening, then there’s a barrier to me saying they can see someone on Monday - I just feel its like too much time.”(Physician 11)

**Table 3:** Participant quotes supporting barriers to treating withdrawal and initiating buprenorphine

1. **Lack of knowledge & experience among the care team** i. “The ones who are a little bit trickier is sometimes we see people who present with the gastrointestinal side effects. So, lots of diarrhea and stomach cramps. Those are cases where I find sometimes it is a bit harder to tease out and they may be in people who are misusing their prescribed opiates.” (Physician 19) ii. “I think generally people are not comfortable and don’t think that prescribing [buprenorphine] is a good idea in an emergency setting because of concern about precipitating withdrawal.” (Physician 4) iii. “The only thing I worry is that, and I have not looked this up myself, is long term safety. So do we know that this is a good long term intervention or the possible harm that we just haven’t encountered yet because we don’t have the numbers or the research for it that we’re going to pick up in five or 10 years.” (Physician 16) iv. “When I ordered it the nurses were almost all like “what’s a COWS”. They had never heard of COWS the first time I did it and that was after it had been going on for a little while.” (Physician 19) v. “[Buprenorphine] was supposed to kind of package it all together and so even though we have suboxone readily available there’s a lot of gray area where it can’t be prescribed. And so then we still don’t know what to do with those patients.” (Physician 9) vi. “Sometimes it was other co-diagnoses like mental health disorders that made it challenging for them to organize the new information. And sometimes it was just someone who had been to our hospital like over 150 times with overdoses and he’s just so unwell that he isn’t able to be open to a new idea.” (Physician 10) vii. most of the time my patients arrive, and it is too short a time frame since their last consumption. By the time we get to the point of having a conversation about taking a prescription of that stuff home quite often they are ready to bolt and they’re not prepared to consider it.” (Physician 1) viii. “If they are on methadone, it’s not a medication we can really give them.” (Physician 10) 2. **Patient disposition and behavior** i. “I would say it’s challenging: rarely an easy interaction, rarely do they understand the consequences of what happened especially if they’ve been given Narcan because they often don’t have a recollection of the events and don’t believe what you’re telling them.” (Physician 16) ii. “The challenge, the biggest point of conflict I find is often when someone should probably stay but they want to go home. We don’t think it’s safe for them to leave. It can be really challenging trying to decide: are they competent to make the decision? Do you restrain them and keep them here?”(Physician 19) iii. “When I refuse to give them [opioids], the first step that they usually go for is “well get somebody else here” … that they want to see another doctor. I will not give them that. Then they’ll say, “well I’m going to complain to the hospital” and … “I’m going to report you to the college” and I’ll say “be my guest”. Then they’ll start calling me names and swearing at me and using foul language and that’s usually when I get security to escort them out.” (Physician 2) iv. “Some people have told me “Look I’m not interested. I’ve tried it and I don’t like it.” (Physician 10) 3. **Logistical constraints** i. “When people come in intoxicated or in withdrawal, they take up a bed for a long time … And so our gut reaction is “we have a busy emergency department and now you’re being demanding of my time and a pain”. It takes time with repeated doses in the emergency department.” (Physician 19) ii. “I don’t think that we can be doing a good job at providing all that counseling ourselves. So given the volumes of patients that we see typically, the length of the interaction with each patient, I don’t think its feasible.” (Physician 14) iii. “They are very high users of the emergency department, and you can put in a little bit of time upfront and decrease their burden on the department.” (Physician 11) iv. “I think ED overcrowding plays a big role in our management of patients in withdrawal … the issue of physical space is not without significance.” (Physician 19) v. “Some people sometimes are reluctant to talk about them in the hallway if they’re in a hallway stretcher because they don’t want everybody else around to hear what their problems are.” (Physician 4) 4. **Initiating a chronic medication in an acute care setting** i. “[Buprenorphine] is really a chronic medication that people will start and continue taking indefinitely. And you know it’s a bit of a philosophical change. I don’t think most ER doctors like the idea of prescribing medications that are used long-term.” (Physician 15) ii. “I think that its akin to saying like do you start blood pressure medications in the emergency department. The patients who are hypertensive. And I have to say that a lot of my colleagues do not because we don’t have a way of following up on those prescriptions.” (Physician 14) iii. “Follow up, repeated prescriptions and … titration of dose would be the other thing. We don’t really aim to provide that in the ER for pretty much any other condition mostly because we don’t intend on seeing you ever again. That’s our philosophy.” (Physician 15) iv. “[Buprenorphine] is not necessarily a medication that I thought would be an emergent use but the more that I see that it can kind of quench some of the significant opioid withdrawal symptoms ... I think once we kind of heard that as a group there was more of an acceptance of using this medication in the emergency department.” (Physician 6) v. “The patients that I have prescribed it for, I’ve had a good response from them initially whether those patients continue to do other follow up and things like that I have no idea.” (Physician 18) vi. “I think the biggest thing is the follow up is unclear always. I mean we initiate these things and we don’t know whether our initiation of them is having an actual positive effect. It’s not really a barrier to our starting it but I guess it’s more if we are starting it, is it definitely helpful or not?” (Physician 3)	

### Variable management of opioid withdrawal

Participants varied in their approach to managing opioid withdrawal and opioid use disorder. Several participants indicated that opioid withdrawal management was frustrating and required nuanced decision-making (Table 1.1i, ii). Many physicians recognized that their approach to medical management and counselling practices likely differed from their colleagues (1.1iii). While some physicians counseled patients extensively on treatment options, harm reduction practices and available resources, others stated that they counselled patients minimally.

Some physicians offered and initiated buprenorphine (1.1iv) while others used non-agonist treatments to target symptoms (e.g. clonidine, non-steroidal anti-inflammatories, anti-diarrheals, benzodiazepines) (1.1v, vi). Participants recognized that opioid medications might be inappropriately prescribed in some cases to patients in opioid withdrawal (1.1 vii). One participant did not feel that there were effective medications for opioid withdrawal (1.1viii). Nevertheless, most physicians interviewed were familiar with buprenorphine and pointed out that its use in the department was increasing, although still in its early stages (1.1ix).

### Facilitators to evidence-based treatment of opioid withdrawal in the ED

#### Departmental champions and support

Physicians frequently stated that their colleagues helped them change their approach to managing opioid withdrawal in the ED including prescribing buprenorphine. These physician champions educated their colleagues about opioid withdrawal and the benefits of buprenorphine (Table 2.1i). Physicians frequently referred to mentorship and informal discussions about their patient cases with more experienced colleagues as important factors in increasing their comfort with prescribing buprenorphine (2.1ii, iii, iv). Some also felt that hearing success stories of patients who had decreased their opioid use after presenting to the ED in withdrawal served as an important motivator to learn more (2.1v).

#### Physician Empowerment & Patient Satisfaction

A few physicians who had initiated buprenorphine in the ED gave accounts of significant patient satisfaction due to resolution of symptoms (Table 2.2i, ii),. These accounts contrasted sjarply with reported experiences using other non-agonist medications which did not resolve symptoms effectively and raised safety concerns (2.2iii). Most physicians with experiences prescribing buprenorphine felt it empowered them to provide a meaningful intervention to a patient population for whom they had no effective options previously (2.2iv).

#### Protocols and order sets

Most ED physicians indicated that having an order set for buprenorphine induction was helpful in the management of opioid withdrawal. The order set prompted physicians to consider prescribing buprenorphine, provided inclusion and exclusion criteria as well as dosing recommendations. Physicians felt that the order set instilled greater confidence and was well-integrated into their workflow (Table 2.3i, ii). The order set is available for reference [see Additional file 2].

#### Timely access to follow-up care

Physicians identified timely follow-up as an important factor in their willingness to initiate buprenorphine in the ED (Table 2.4i). Physicians felt that the follow-up should be available the next day, though some were comfortable if patients could access it within the next few days (2.4ii). Limited follow-up during the weekend reduced their comfort with prescribing buprenorphine in some cases (2.4iii).

### Barriers to evidence-based treatment of opioid withdrawal

#### Lack of experience and confidence among the care team

Most physicians still felt inexperienced with managing opioid withdrawal, with some concerned about missing subtle presentations (Table 3.1i) and the risk of precipitating withdrawal when initiating buprenorphine therapy (Table 3.1ii). Others noted uncertainty about the long-term safety of buprenorphine as a barrier to prescribing (Table 3.1iii). Many physicians hesitated to use the opioid withdrawal order set as the nursing staff lacked familiarity with buprenorphine and the Clinical Opiate Withdrawal Scale (COWS) (Table 3.1iv).

Some physicians who were interested in prescribing buprenorphine experienced challenges when they encountered more complex cases (3.1.v). Factors such as medical or psychiatric comorbidities (3.1vi) or a patient’s lack of interest in treatment (3.1vii) presented additional challenges. Some physicians encountered difficulties with buprenorphine induction for patients who used methadone or had recently used other opioids (3.1vii, viii).

#### Negative patient interactions

In some cases, physicians had experienced negative interactions with patients which affected their motivation to counsel and treat OUD in the ED. Physicians spoke of cases of aggressive behaviours which made for stressful encounters (3.2i). Tensions between patient and physician arose in cases where the patient wanted to leave after an overdose but the physician felt it was unsafe (3.2ii) or when the patient requested an opioid medication which the physician did not believe was indicated `3.2iii). These adversarial interactions limited the establishment of rapport and presented a barrier to further counselling and discussion of buprenorphine therapy. In some cases, patients declined due to previous poor experiences with the medication (3.2iv).

#### Logistical constraints

Physicians commonly cited time constraints as a barrier to treating opioid withdrawal in the ED. Some physicians identified waiting for the patient to reach an appropriate level of withdrawal and subsequent monitoring upon initiating the medication as barriers (Table 3.3i). Concerns around time requirements for proper counselling were common (3.3ii). Some physicians disagreed that time constraints were a barrier to the management of withdrawal and rather focused on the potential to reduce future visits by intervening (3.3iii).

The physical environment of the ED was cited as an important barrier to comprehensive management of opioid withdrawal, including overcrowding (3.3iv), “hallway medicine” and a lack of privacy (3.3v). Physicians felt that both time and physical constraints made extensive counselling difficult to take on in the ED.

#### Initiating a chronic medication in an acute care setting

Some physicians did not feel that prescribing a long-term medication in an ED setting was appropriate (Table 3.4i) with comparisons to hypertension and hyperlipidemia management (3.4ii). The need for medication titration, uncertainty of immediate follow-up and long-term management were reported (3.4iii). Some perspectives changed when they considered the potential of this medication to effectively address opioid withdrawal in the acute setting (3.4iv).

Furthermore, many physicians felt uncertain about the effectiveness of their intervention in managing opioid withdrawal. Those that referred patients to the outpatient follow-up clinic identified that they had no feedback on whether the patient had attended (3.4v). Most felt that it would be helpful to know if patients were attending (3.4vi).

## Discussion

This is a qualitative study describing barriers and facilitators to addressing OUD and prescribing buprenorphine in an urban Canadian ED setting. The Canadian guidelines recommend buprenorphine as first-line for management of OUD [3]. Our findings suggest highly variable approaches to managing opioid withdrawal and the need for a targeted knowledge translation strategy to address common barriers and shift ED culture to one that celebrates treatment of acute presentations to affect long-term outcomes. In Canada, there are no regulatory barriers or exemption programs needed in order to prescribe buprenorphine [24].

Most physicians reported limited or no experience prescribing buprenorphine and expressed feelings of dissatisfaction and frustration with their current interventions for OUD in the ED. Similar perspectives have been reported in a recent survey of ED clinicians in a tertiary center in the United States [18]. A key difference though was that a minority of the interviewees in that study were in favor of ED-initiated buprenorphine whereas most participants in our study agreed that it should be considered [18]. In our study, physicians reported more favorable attitudes with increased clinical education and exposure. In both studies, respondents agreed that ED-initiated buprenorphine was feasible if appropriate system supports were implemented.

Our findings align with previous research showing that emergency physicians perceive that system level facilitators including order sets and departmental protocols increase their uptake of procedures in the ED [16, 25]. Our recent Canada-wide survey of ED physicians also highlighted the potential value of order sets in shifting culture and practice [26]. Despite the availability of these resources, participants suggested that additional guidance around challenging cases would be helpful as part of the knowledge translation strategy, including complexities around polysubstance withdrawal, comorbidities, and relative contraindications to buprenorphine induction.

There is also a role for mentorship, continuing education, and additional clinical exposure. Our study found that a significant facilitator to adoption of buprenorphine in the ED was the presence of physician champions who led departmental change, consistent with literature which shows that role modeling builds physician comfort with new practices [27, 28]. Having outpatient clinics available addresses concerns of initiating a chronic medication in an acute setting, titrating the dose, and providing ongoing care. Rapid access clinics demonstrate positive clinical outcomes and retention in care, forming a crucial component of ED-initiated treatment for OUD [9, 29]. ED leadership should work closely with outpatient clinics to ensure rapid follow-up and a smooth transition of care from the ED to the outpatient clinic.

Our study identified persistent perceived barriers to buprenorphine use in an academic ED which had already initiated these supports including order sets, training, and follow up for patients. Additional barriers reported included lack of patient interest, discomfort with counselling around buprenorphine, lack of nursing support, time constraints and safety concerns as perceived barriers for ED physicians [16–18].. These findings highlight the need for earlier, more robust education for ED physicians and nurses on the use of buprenorphine.

Various frameworks suggest that guidelines are not adopted due to either knowledge, attitude or behaviours [30–34]. We found all three types of barriers in our findings; physicians reported lack of knowledge, uncertainty about the impacts of using buprenorphine for opioid withdrawal as well as environmental and patient factors as barriers to uptake. Treatment of OUD presents unique challenges in the ED given frequently associated social and medical complexities.

Additionally, the treatment of OUD is often stigmatized by physicians which can impact clinical outcomes [35, 36] and hinder knowledge translation [37]. Future studies should further explore the relationship between stigma and attitudes and perceived barriers to treating OUD.

Departments should develop a knowledge translation and implementation strategy that directly addresses perceived barriers in order to optimize guideline adoption [38]. Future research may also focus on understanding perceived barriers of health care administrators and allied health care staff in supporting buprenorphine prescribing in the ED setting [32]. These findings could inform our knowledge translation strategies, as we suspect from this study that early involvement of allied health professionals, including nursing and social work, in buprenorphine counselling would vastly improve uptake.

### Limitations

This qualitative study had some important limitations. We interviewed physicians from one organization at one point in time which limits the generalizability of our findings. Generalizability is further limited by constructivist grounded theory methodology which considers the unique context and experiences of participants in shaping their reality which may vary considerably among emergency physicians. Although we randomly invited physicians, only half of physicians in the department were invited to participate to ensure that all interviews could be completed during the study period. Participants self-selected to participate in interviews which introduced some selection bias. Inviting all emergency physicians in the department could have improved sample variation. Finally, although participants were assured of confidentiality, we cannot exclude the possibility of socially desirable responses.

## Conclusion

This study describes barriers and facilitators to addressing OUD and prescribing buprenorphine in a Canadian ED setting. Our findings suggest highly variable practices and the need for a targeted knowledge translation strategy to address common perceived barriers. Additional research is needed to elucidate perceived barriers of health care administrators and allied health care staff in supporting buprenorphine prescribing in the ED setting. Our findings suggest a role for physician champions, involvement of allied health professionals in counseling, and expanded education.

## Supplementary Information

**Additional file 1.**

**Additional file 2.**

## Data Availability

The datasets generated and/or analysed during the current study are not publicly available due to participant confidentiality but are available from the corresponding author on reasonable request.

## Abbreviations

OUD: opioid use disorder
ED: emergency department
