# Supplementary material for: A qualitative examination of the current management of opioid use disorder and barriers to prescribing buprenorphine in a Canadian emergency department
Source: BMC Emerg Med. 2021 Apr 15;21:48. doi: 10.1186/s12873-021-00443-1 (PMC8051038; doi:10.1186/s12873-021-00443-1)
Supplement: Supplementary file 1 — Additional file 1. [file 12873_2021_443_MOESM1_ESM.docx]

**Interview Guide**

***Brief Introduction***

***Clinician’s experience with the patient population***

- Please describe your experience treating patients in the ED who have overdosed or use opioids.
  - How much experience
  - Describe How have you found interacting with these patients
- Please describe your experience treating patients who are in opioid withdrawal in the ED.

***Perceived role of the ED in supporting addiction treatment***

- What do you think is the role of the ED in addressing opioid addictions?
  - What parts of this are within the scope of practice of ED physicians?
- What do you think is the role of the ED in preventing future overdoses?
  - What parts of this are within the scope of practice of ED physicians?
- What do you think is the role of the ED in education around opioid use – for example, counselling around treatment options, or harm reduction?
  - What parts of this are within the scope of practice of ED physicians?
- How do your colleagues’ manage opioid addiction in the ED?
  - Do they prescribe suboxone in the ED? What do you think are your colleagues’ practices and beliefs about prescribing suboxone in the ED?
  - What are other practices you have seen in the ED related to opioid addiction?
    - (medication prescriptions?)

***Clinician’s experience with suboxone***

- Have you ever prescribed suboxone or have you every seen it prescribed?
  - If have not prescribed: Are you interested in using it? Why or why not?
  - If have prescribed: What were your experiences with prescribing suboxone? How did it go?
- What resources would you need to prescribe it?
- What training have you had in prescribing suboxone?
- What are the challenges to prescribing suboxone in the ED?
  What solutions can you suggest that might help overcome these challenges?

***Demographic Information***

- Gender
- EM Training (CFPC-EM vs. FRCP)
- Years in practice in ED
- Type of practice (ED only, Mixed Practice)
