# Supplementary material for: A qualitative examination of the current management of opioid use disorder and barriers to prescribing buprenorphine in a Canadian emergency department
Source: BMC Emerg Med. 2021 Apr 15;21:48. doi: 10.1186/s12873-021-00443-1 (PMC8051038; doi:10.1186/s12873-021-00443-1)
Supplement: Supplementary file 2 — Additional file 2. [file 12873_2021_443_MOESM2_ESM.docx]

|  | **Prescriber Instructions:**   1. Assess for indications for {SUBOXONE} buprenorphine-naloxone:   Clinical Opiate Withdrawal Scale (COWS) greater than 12 and each of the following conditions are met:   - - **At least** 12 hours since last **short acting** opioid (e.g. fentanyl buccal / sublingual / IV / smoked / snorted; heroin, HYDROmorphone, morphine, oxycodone) **OR** crushed and/or tampered long acting opioid   - **At least** 24 hours since last **long acting** opioid (e.g. HYDROmorphone CR (controlled release), Oxycodone CR (controlled release), morphine ER (extended release), fentanyl transdermal patch)   Note: long acting formulations become short acting agents if crushed and/or tampered with   - - **At least** 72 hours since last methadone dose  1. Assess for contraindications:  - Allergy or hypersensitivity to buprenorphine or naloxone - Severe liver dysfunction - Acute severe respiratory distress - Decreased level of consciousness - Patient unable to provide informed consent - Suspected ileusechanical obstruction   If patient declines {SUBOXONE} buprenorphine-naloxone therapy, consider supportive management as outlined below. Provide education on loss of tolerance and risk of overdose with rapid detoxification. | | |
| --- | --- | --- | --- |
|  |  | Name of opioid last used: _ _ _ _ _ _ _ _ _ _ _ _ _ _ Date/Time: _ _ _ _ _ _ _ _ _ _ _ _ _ _ | |
|  |  | Name of opioid last used: _ _ _ _ _ _ _ _ _ _ _ _ _ _ Date/Time: _ _ _ _ _ _ _ _ _ _ _ _ _ _ | |
|  |  | Name of opioid last used: _ _ _ _ _ _ _ _ _ _ _ _ _ _ Date/Time: _ _ _ _ _ _ _ _ _ _ _ _ _ _ | |
|  |  | Name of opioid last used: _ _ _ _ _ _ _ _ _ _ _ _ _ _ Date/Time: _ _ _ _ _ _ _ _ _ _ _ _ _ _ | |
|  |  | 1. **MONITORING:** |  |
|  | 🗹 | Vital Signs: Temperature, HR, RR, BP, and O_2_ saturation q2h and as needed |  |
|  |  | **Notify MRP if:** |  |
|  |  | O_2_ saturation less than 92% and/or RR less than 10 breaths per minute |  |
|  | 🗹 | Clinical Opioid Withdrawal Scale (COWS) at presentation and q2h |  |
|  |  |  |  |
|  | 🗹 | Discontinue COWS assessment when COWS less than 5 or maximum dose of {SUBOXONE} buprenorphine-naloxone reached |  |
|  |  | **Notify MRP if:** |  |
|  |  | COWS less than 5 or when maximum dose of {SUBOXONE} buprenorphine-naloxone has been administered |  |

|  |  | 1. **LABORATORY TESTS:**   **Note to Prescriber:** Do not delay treatment while waiting for investigations. |  |
| --- | --- | --- | --- |
|  | 🞎  **OR**  🞎 | Urine pregnancy test  HCG, pregnancy screen |  |
|  | 🞎 | Urine drugs of abuse screen |  |
|  | 🞎 | CBC, electrolytes (sodium, potassium, chloride), creatinine, glucose, AST, ALT, ALP, total bilirubin |  |
|  |  |  |  |
|  |  | **3. MEDICATIONS:** |  |
|  |  | **DAY ONE: Initial 24 hours** |  |
|  |  | 1. **STANDARD DOSING** |  |
|  | 🞎 | {SUBOXONE 2-0.5} buprenorphine 2 mg-naloxone 0.5 mg  2 tablets sublingual once for COWS greater than 12  Observe patient until {SUBOXONE} buprenorphine-naloxone is fully dissolved under the tongue (usually within 2 to 10 minutes) |  |
|  |  | 1. **IF PATIENT AT RISK OF RESPIRATORY DEPRESSION, CNS DEPRESSION, OR ELDERLY (GREATER THAN 65 YEARS), ORDER:** |  |
|  | 🞎 | {SUBOXONE 2-0.5} buprenorphine 2 mg-naloxone 0.5 mg  1 tablet sublingual once for COWS greater than 12 |  |
|  |  | Observe patient until {SUBOXONE} buprenorphine-naloxone is fully dissolved under the tongue (usually within 2 to 10 minutes) |  |
|  |  | **DAY ONE SUBSEQUENT DOSES:** |  |
|  | 🗹 | COWS score q2h |  |
|  | 🗹 | Notify MRP if COWS score increases after {SUBOXONE} buprenorphine-naloxone given |  |
|  | 🞎 | 1. **STANDARD DOSING**   {SUBOXONE 2-0.5} buprenorphine 2 mg-naloxone 0.5 mg  2 tablets sublingual q2h for COWS greater than 5, max 3 additional doses in 24 hours (maximum total 16 mg of buprenorphine component in 24 hours)  Observe patient until {SUBOXONE} buprenorphine-naloxone is fully dissolved under the tongue (usually within 2 to 10 minutes)  **B) IF PATIENT AT RISK OF RESPIRATORY DEPRESSION, CNS DEPRESSION, OR ELDERLY (GREATER THAN 65 YEARS), ORDER:** |  |
|  | 🞎 | {SUBOXONE 2-0.5} buprenorphine 2 mg-naloxone 0.5 mg  1 tablet sublingual q2h for COWS greater than 5, max 7 additional doses in 24 hours (maximum total 16 mg of buprenorphine component in 24 hours)  Observe patient until {SUBOXONE} buprenorphine-naloxone is fully dissolved under the tongue (usually within 2 to 10 minutes) |  |
|  |  | **DAY TWO:** |  |
|  | 🗹 | COWS score in AM |  |
|  | 🗹 | Calculate total {SUBOXONE} buprenorphine-naloxone dose received on Day 1. Administer this dose in AM of Day 2. |  |
|  | **AND** |  |  |
|  | 🗹 | {SUBOXONE 2-0.5} buprenorphine 2 mg-naloxone 0.5 mg  1 to 2 tablets sublingual for 1 dose in AM of Day 2 if COWS greater than 5 (maximum total 20 mg of buprenorphine component on Day 2) |  |
|  | 🗹 | **DAY THREE:** COWS score in AM |  |
|  | 🗹 | Calculate total {SUBOXONE} buprenorphine-naloxone dose received on Day 2. Administer this dose in AM of Day 3 |  |
|  | **AND** |  |  |
|  | 🗹 | {SUBOXONE 2-0.5} buprenorphine 2 mg-naloxone 0.5 mg  1 to 2 tablets sublingual for 1 dose in AM of Day 3 if COWS greater than 5 (maximum total 24 mg of buprenorphine component on Day 3) |  |
|  | 🗹 | **DAY FOUR AND BEYOND:**  COWS score in A.M. |  |
|  |  | **Note to Prescriber:**  MRP to write new order for maintenance {SUBOXONE} buprenorphine-naloxone equal to Day 3 total buprenorphine dose if symptoms well controlled or maximum dose of 24 mg of buprenorphine reached, and discontinue Opioid Withdrawal Order set  **OR**  MRP to write new order to continue titration of {SUBOXONE 2-0.5} buprenorphine 2 mg-naloxone 0.5 mg 1 to 2 tablets sublingual for COWS greater than 5 until maximum dose of 24 mg of buprenorphine reached |  |
|  |  | **SUPPORTIVE MANAGEMENT OF WITHDRAWAL SYMPTOMS:**  **Note to Prescriber:**  Consider supportive management if maximum daily dose of {SUBOXONE} buprenorphine-naloxone reached **OR** patient declines {SUBOXONE} buprenorphine-naloxone therapy, **AND** ongoing withdrawal symptoms |  |
|  | 🗹 | acetaminophen 1,000 mg oral q6h as needed for pain, max 4g in 24 hours  NOTE: Total daily dose of acetaminophen from all sources must not exceed 4g |  |
|  | 🗹 | ibuprofen 400 mg oral q6h as needed for pain |  |
|  | 🗹 | clonidine 0.1 mg oral BID as needed for sweating, yawning, restlessness, anxiety / irritability, or gooseflesh skin |  |
|  | 🗹 | loperamide 4 mg oral for 1 dose as needed for diarrhea, THEN 2 mg oral as needed after each loose bowel movement, max 16 mg in 24 hours |  |
|  | 🞎 | ondansetron 8 mg oral q4h as needed for nausea, max 16 mg in 24 hours |  |
|  | **OR** |  |  |
|  | 🞎 | ondansetron 4 mg IV q4h as needed for nausea, max 16 mg in 24 hours |  |
|  |  |  |  |
|  |  | 1. **DISCHARGE INSTRUCTIONS:** |  |
|  | 🗹 | Provide patient with outpatient prescription for {SUBOXONE} buprenorphine-naloxone, completed by MD (see last page for instructions) |  |
|  | 🗹  🗹  🗹  🗹 | Fax referral to Rapid Access Addiction Medicine (RAAM) clinic  Provide patient with education on where to obtain a Take Home Naloxone Kit  Provide patient with RAAM handout and instruct patient to drop in to RAAM clinic (see last page for instructions)  Review harm reduction practices as outlined on RAAM handout |  |
|  |  |  |  |
|  | \|  \|  \|  \|  \| \| --- \| --- \| --- \| --- \| \| Print Name and Designation \| Prescriber`s Signature \| Date (dd/mm/yy) \| Time \| \| Phone/ Pager: _ _ _ _ _ _ _ _ _ _ _ \| \|  \|  \| | | |

**Prescription Instructions for Providers:**

Provide patient with prescription for {SUBOXONE} buprenorphine-naloxone for the days between discharge from hospital and the first appointment with the RAAM clinic.

The outpatient prescription should include:

- Direction to community pharmacy to observe daily dose, to a maximum of 7 days of treatment. Further doses to be prescribed by community providers.
- Last dose {SUBOXONE} buprenorphine ___mg-naloxone___mg AND date/time {SUBOXONE} buprenorphine-naloxone administered in hospital AND location administered.
- Start date: the day following the day of patient discharge from hospital
- End date (inclusive): the day of the first appointment with the RAAM clinic
- Daily dose: the total amount received in hospital in the last 24 hours

**Example**:

{SUBOXONE} buprenorphine-naloxone _____mg-_____ mg SL once daily

from __________ (date) to __________ (date) inclusive. Observe daily dose.

Last dose buprenorphine_____mg-naloxone_____ mg given on __________________

(date/time) at ____________________(name) unit in ______________________ (name)

hospital.
